# Serum TGF-β1 and SMAD3 levels are closely associated with coronary artery disease

**DOI:** 10.1186/1471-2261-14-18

**Published:** 2014-02-17

**Authors:** Can Chen, Wei Lei, Wenjiang Chen, Jianfeng Zhong, Xiaoxin Gao, Bo Li, Huailong Wang, Congxin Huang

**Affiliations:** 1 Department of Cardiovascular Medicine, Renmin Hospital of Wuhan University, Wuhan 430060, China; 2 Department of Cardiovascular Medicine, The Affiliated Hospital of Guangdong Medical College, Zhanjiang 524000, China

**Keywords:** Coronary artery disease, TGF-β1, SMAD3 protein, Human, Biomarkers

## Abstract

**Background:**

Coronary artery disease (CAD) is one of the most common diseases leading to mortality and morbidity worldwide. There is considerable debate on whether serum transforming growth factor β1 (TGF-β1) levels are associated with long-term major adverse cardiovascular events in patients with CAD, and to date, no study has specifically addressed levels in patients with different degrees of CAD severity.

**Methods:**

Serum TGF-β1 and mothers against decapentaplegic homolog 3 (SMAD3) concentrations were evaluated in 279 patients with CAD and 268 controls without CAD. The clinical and biochemical characteristics of all subjects were also determined and analyzed.

**Results:**

TGF-β1 and SMAD3 concentrations in CAD patients were significantly higher than those in the controls. The serum TGF-β1 level in acute myocardial infarction (AMI) was significantly higher than that in both stable angina pectoris (SAP) and unstable angina pectoris (UAP) (p < 0.05), while there was no marked difference between levels in SAP and UAP (p > 0.05). SMAD3 levels showed no obvious difference among AMI, SAP, and UAP. TGF-β1 and SMAD3 are potential biomarkers for CAD, and may be more accurate than Lpa, ApoA1, uric acid, BUN, or triglycerides (TG).

**Conclusions:**

Serum TGF-β1 and SMAD3 levels are closely associated with CAD, and may become useful biomarkers for diagnosis and risk stratification.

## Background

Coronary artery disease (CAD) is one of the leading causes of mortality and morbidity worldwide, especially in many developed countries [1,2]. According to the World Health Organization, it is predicted that 23.6 million deaths per year will be because of cardiovascular diseases by 2030 [3]. CAD, with a complex etiology, is considered to be the result of an interaction between genetic and environmental factors [4-6]. In the past decades, many contributing factors including smoking, hypertension, diabetes mellitus (DM), atherosclerosis, obesity, and diet have been established, but the exact etiology underlying CAD remains obscure. Increasing evidence suggests that inflammation plays an important role in the pathogenesis of CAD [7,8].

A number of inflammatory cytokines mediate adverse cardiovascular events in patients with CAD, and transforming growth factor-β1 (TGF-β1) has garnered particular attention because of its multiple roles in important pathological changes such as enhancement of macrophage and fibroblast chemotaxis, stimulation of extracellular matrix (ECM) synthesis, and vascular cell proliferation abnormalities [9,10]. Although it has been known that TGF-β1 activates several pathways, including the extracellular-regulated kinase pathway, the nuclear factor-κB pathway, and the phosphatidylinositol-3-kinase pathway [11,12], its main signaling mechanism is linked to the mothers against decapentaplegic homolog 3 (SMAD3) family [13]. TGF-β1 binds to its type I receptors and forms a heteromeric complex with the type II receptor, which subsequently results in phosphorylation and activation of the TGF-β1 downstream signaling molecules SMAD2 and SMAD3. Activated SMAD2 or SMAD3 heterodimerizes with SMAD4 and then introduces the above complex into the nucleus, where it regulates the expression of its target gene [14,15]. TGF-β1/SMAD3-dependent pathways play a pivotal role in mediating different biological effects of TGF-β1 such as cell proliferation, immune suppression, and inflammation [12,16-18]. In the cardiovascular system, myocardial TGF-β1 expression is markedly activated in patients with hypertrophic or dilated cardiomyopathy, and in experimental models of myocardial hypertrophy and myocardial infarction [19]. Other studies suggest that TGF-β signaling may be crucial for repression of inflammatory gene expression in healing infarcts mediated by an inflammatory infiltrate [20]. TGF-β1 is crucial in the pathogenesis of infarct healing, cardiac remodeling, and interstitial fibrosis. There have been few clinical investigations of TGF-β1/SMAD3 signals in large patient groups. There is considerable debate over any correlation of TGF-β1 levels with major adverse cardiovascular events in patients with CAD. In particular, no study has specifically addressed patients with different degrees of CAD [9].

We collected serum samples from 279 patients with CAD and from 297 controls, and assessed for any association between serum TGF-β1/SMAD3 levels and the presence and severity of CAD. We evaluated the potential clinical application of measurements of these cytokines for CAD diagnosis, comparing their utility with the currently used biochemical indicators.

## Methods

### Study population

Written informed consent was obtained from the population involved in this study and the study protocol was approved by the Ethics Committee of Affiliated Hospital of Guangdong Medical College.

A total of 279 patients who were referred for coronary angiography by their attending physicians from May to August 2013, and who were found to have at least 50% stenosis in at least one coronary artery, were included. Their ages were about 69.30 years. The 268 controls were consecutive subjects undergoing routine medical examinations at the Physical Examination Center. Those who had any clinical manifestations or a medical history of heart disorders, a family history of coronary artery disease, or abnormal ECG were excluded. Exclusion criteria also included acute or chronic infections or inflammatory diseases, severe hepatic or renal dysfunction, malignant tumors, or hematologic disorders. The CAD group was divided into three subgroups: acute myocardial infarction (AMI), unstable angina pectoris (UAP), and stable angina pectoris (SAP).

### Physiological evaluation and blood collection

All study participants underwent a standard clinical examination. Body mass index (BMI) was calculated as the individual's body mass divided by the square of their height. Smoking was defined as having smoked for at least 1 year currently. Hypertension was defined as a systolic blood pressure of ≥ 140 mm Hg or use of antihypertensive therapy. Diabetes was diagnosed according to nonfasting glucose levels ≥ 11.1 mmol/L.

Approximately 2 mL of venous blood was collected from each subject, and then kept in tubes without preservatives at 4°C overnight. Serum aliquots were obtained after centrifugation at 1000 g for 40 min at 4°C.

### Coronary angiography

All coronary angiographies were performed using standard technique. Significant CAD was considered to be present if there was an internal luminal stenosis ≥ 50% in the left main coronary artery, right coronary artery, and/or their major branches. The degree of coronary atherosclerosis was further categorized according to the number of coronary vessels with significant stenoses as 1-, 2-, or ≥ 3-vessel disease. All catheterizations and imaging analyses were performed by two experienced interventional cardiologists who were unaware of the patients’ clinical data.

### Biochemical assay

Fasting blood samples were obtained and prepared for the biochemical assay of clinical indices associated with CAD diagnosis, including lipoprotein (a) (Lp(a)), blood urea nitrogen (BUN), uric acid (SUA), blood glucose (Glu), total cholesterol (CHOL), high-density lipoprotein (HDL), low-density lipoprotein (LDL), triglycerides (TG), apolipoprotein A1 (ApoA1), and apolipoprotein B (ApoB).

### Measurements of TGF-β1 and Smad3 levels

Determination of serum active TGF-β1 and SMAD3 concentration was performed by ELISA using the ELISA-Quantikine kit (R&D Systems, Minneapolis, MN, USA) according to the manufacturer’s instructions. The detection range of the ELISA used to measure TGF-β1 was 100–1200 ng/L, and the detection limit of SMAD3 was 2.5–30 pg/mL. Their accuracy was expressed in the correlation coefficient, which was required to be greater than 0.9999.

### Statistical analysis

Categorical and continuous variables were expressed as percentiles and mean ± standard error of mean (SEM), respectively. Differences among groups were assessed using the chi-squared or analysis of variance tests, followed by post-hoc analysis (Bonferroni’s correction) for comparison among groups. The association between serum TGF-β1 or SMAD3 and CAD or its risk factors was estimated using a multivariate logistic regression model.

## Results

### Patient characteristics

The main demographic and clinical characteristics of all the studied subjects are summarized in Table 1.

**Table 1:** Clinical and biochemical characteristics of the study population

**Variables**	**CAD**	**Control**	**p**
Sex (M/F)	168/111	180/88	0.0913
Age (years)	69.30 ± 0.7049	63.73 ±0.6952	< 0.0001
BMI (kg/m^2^)	23.18 ±0.1337	21.04 ±0.08991	<0.0001
Smoke (%)	12.19	5.60	0.007
Hypertension (%)	26.16	18.66	0.0355
DM (%)	25.81	15.30	0.0024
AF (%)	8.60	0	< 0.0001
Stroke (%)	7.17	1.12	0.0004
High TC (%)	10.39	8.21	0.3796
High TG (%)	13.62	7.09	0.0125
Low HDL (%)	28.32	38.06	0.0155
High LDL (%)	10.04	9.33	0.7798
Lp(a) (mg/l)	265.4 ± 12.43	215.9 ± 11.11	0.0033
BUN (mmol/l)	5.898 ± 0.1978	5.075 ± 0.1675	0.0016
SUA (μmol/l)	340.6 ± 6.018	280.6 ± 6.629	< 0.0001
Glu (mmol/l)	6.549 ± 0.1418	6.011 ± 0.1231	0.0046
CHOL (mmol/l)	4.785 ± 0.07130	4.821 ± 0.2581	0.8885
TG (mmol/l)	1.567 ± 0.07598	1.341 ± 0.04895	0.0148
HDL (mmol/l)	1.256 ± 0.0214	1.242 ±0.02509	0.6613
LDL (mmol/l)	2.854 ± 0.05867	4.190 ± 1.467	0.3377
ApoA1 (g/l)	1.339 ± 0.02123	1.262 ± 0.02484	0.0187
ApoB (g/l)	0.9414 ± 0.01682	0.8810 ± 0.01690	0.0117

There were significant differences between CAD and control groups in important risk factors including age, BMI, smoking, hypertension, DM, atrial fibrillation (AF), stroke, TG, HDL, Lp(a), BUN, uric acid, ApoA1, and ApoB. CAD patients were divided into three subgroups by pathological types: AMI, SAP, and UAP (Table 2). The statistical analysis revealed that the risk factors of CAD mentioned above were significantly higher than in the controls, and, additionally, several indices, including BMI, AF, uric acid, total cholesterol, and ApoA1 showed significant differences among the three subgroups. Different levels of total cholesterol were found in acute myocardial infarction and angina pectoris patients, but not in CAD and control subjects.

**Table 2:** Clinical and biochemical characteristics of the study population with differing CAD severity

**Variables**	**CAD**			**Control**
	**AMI**	**SAP**	**UAP**	
Sex (M/F)	30/21	92/69(M/F)	46/21(M/F)	180/88(M/F)
Age (years)	67.86 ±1.823	69.97 ±0.8970	68.81 ± 1.438	63.73 ±0.69***
BMI (kg/m^2^)	22.72 ± 0.2705	23.08 ± 0.1743#	23.78 ± 0.2934#	21.04 ±0.089***
Smokers (%)	15.69	11.18	11.94	5.60*
Hypertension (%)	31.37	21.12	10.35	18.66***
DM (%)	27.45	27.33	20.90	15.30**
AF (%)	13.72	3.73#	1.49#	0***
Stroke (%)	7.84	5.59	10.45	1.12***
High TC (%)	19.61	8.07	8.96	8.21
High TG (%)	17.68	11.80	14.92	7.09*
Low HDL (%)	15.69	30.43	32.84	38.06**
High LDL (%)	13.72	9.32	8.96	9.33
Lp(a) (mg/l)	292.5 ± 33.46	244.4 ± 14.90	295.3 ± 27.06	215.9 ± 11.11**
BUN (mmol/l)	6.424 ± 0.5315	5.628 ± 0.2592	6.147 ± 0.3538	5.075 ± 0.167**
SUA (μmol/l)	364.4 ± 11.62	328.2 ± 8.385#	352.2 ± 11.56#	280.6 ± 6.629***
Glu (mmol/l)	6.555 ± 0.3058	6.658 ± 0.1950	6.281 ± 0.2754	6.011 ± 0.1231*
CHOL (mmol/l)	5.190 ± 0.1761	4.695 ± 0.09342#	4.690 ± 0.1346#	4.821 ± 0.2581
TG (mmol/l)	1.624 ± 0.1808	1.528 ± 0.1039	1.619 ± 0.1391	1.341 ± 0.04895
HDL (mmol/l)	1.322 ± 0.04794	1.240 ± 0.02690	1.246 ± 0.04980	1.242 ±0.02509
LDL (mmol/l)	3.134 ± 0.1226	2.798 ± 0.07992	2.773 ± 0.1157	4.190 ± 1.467
ApoA1 (g/l)	1.449 ± 0.05356	1.308 ± 0.02764#	1.329 ± 0.04026#	1.262 ± 0.02484**
ApoB (g/l)	1.003 ± 0.04239	0.9385 ± 0.0322	0.9414 ± 0.0168	0.8810 ± 0.01690*

Date are means ± SEM or n (%).

#p < 0.05 vs SAP or UAP and *p < 0.05, **p < 0.01, ***p < 0.001 vs Control.

### TGF-β1 and SMAD3 levels in CAD patients

**Figure 1:**
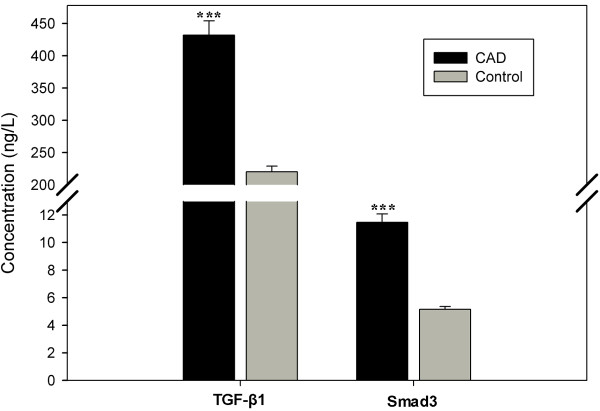
Serum TGF-β1 and SMAD3 levels from CAD patients, ***p < 0.0001, *p < 0.05.

TGF-β1 and SMAD3 levels were both increased significantly in the CAD group compared with the controls (p < 0.0001) (Figure 1). Serum TGF-β1 and SMAD3 levels in the AMI, SAP, and UAP subgroups were all higher than in the control group (p < 0.0001) (Figure 1), suggesting that the two cytokines were related to CAD pathogenesis.

**Figure 2:**
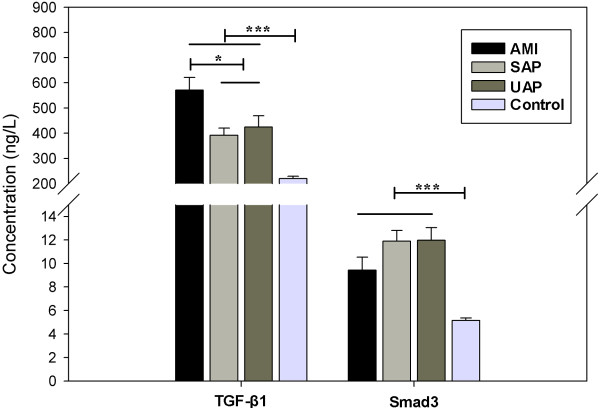
Serum TGF-β1 and SMAD3 levels from patients with different CAD severity, ***p < 0.0001, *p < 0.05.

Comparing among the three subgroups of CAD subjects, serum TGF-β1 levels in AMI were significantly higher than those in both SAP and UAP (p < 0.05), while there was no marked difference between those in SAP and UAP (p > 0.05) (Figure 2). However, SMAD3 levels were not significantly different among AMI, SAP, and UAP subgroups, suggesting that TGF-β1 may regulate the progression from angina pectoris to AMI.

### Correlation of TGF-β1 and SMAD3 levels with CAD occurrence and its risk factors

**Figure 3:**
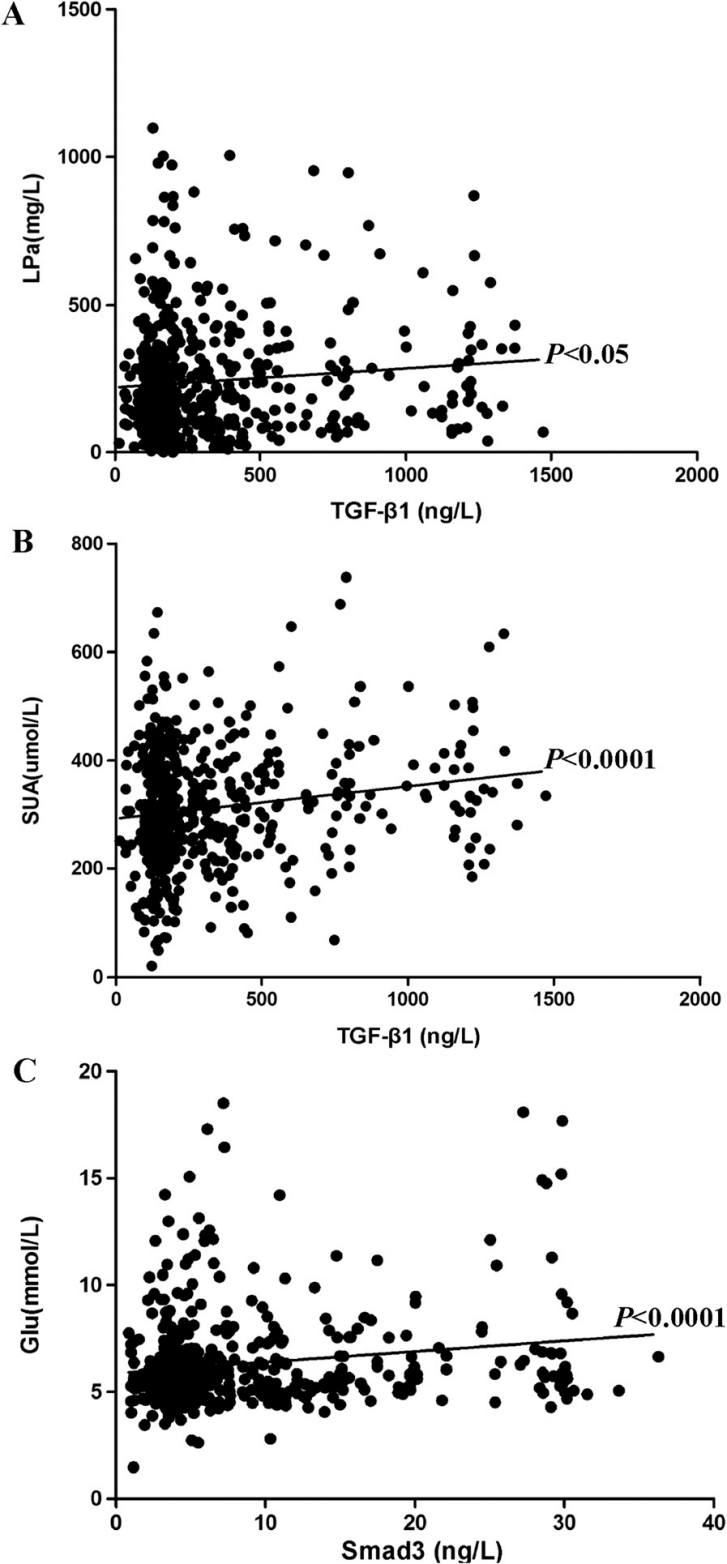
**Correlation of TGF-β1 or SMAD3 with the risk factors of CAD, (A) Correlation analysis of TGB- β 1 with LPa; (B) Correlation analysis of TGB- β with SUA; (C) SMAD3 with Glu.** ***p < 0.0001, *p < 0.05.

To explain a potential mechanism of TGF-β1 and SMAD3, we evaluated their relationship with CAD and its risk factors including Lp(a), BUN, creatinine, uric acid, blood glucose, total cholesterol, TG, HDL, LDL, ApoA1, and ApoB. There was a very significant correlation between CAD and concentration of TGF-β1 and SMAD3 (p < 0.0001). Among the other biochemical indices [21], TGF-β1 significantly correlated with Lp(a) (p < 0.0203) and uric acid (p < 0.0001), respectively, and SMAD3 correlated very significantly with blood glucose (p < 0.0001) (Figure 3).

### TGF-β1 and SMAD3 as biomarkers for CAD

**Figure 4:**
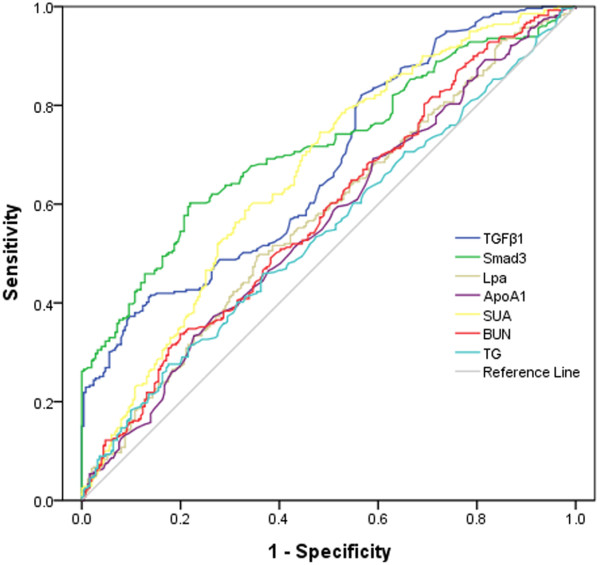
**Receiver operating characteristic (ROC) curves for TGF-β1 and SMAD3.** The area under the curve of TGF-β1 was 0.678 (95% confidence interval [CI], 0.633–0.723). The area under the curve of SMAD3 was 0.715 (95% CI, 0.672–0.758).

Far higher TGF-β1 and SMAD3 concentrations occurred in CAD patients (TGF-β1: 432.2 ± 22.12 ng/L; SMAD3: 11.47 ± 0.6161 ng/L) compared with controls (TGF-β1: 220.1 ± 8.831 ng/L; SMAD3: 5.157 ± 0.1965 ng/L). The area under the Receiver operating characteristic curves (AUC_ROC_) indicated that both TGF-β1 (AUC_ROC_: 0.678, 95% confidence interval [CI]: 0.633–0.723) and SMAD3 (AUC_ROC_: 0.715, 95% CI: 0.672–0.758) were the more potent biomarkers for CAD, compared with Lpa (AUC_ROC_: 0.574, 95% CI: 0.525–0.623), ApoA1 (AUC_ROC_: 0.561, 95% CI: 0.512–0.609), uric acid (AUC_ROC_: 0.663, 95% CI: 0.617–0.710), BUN (AUC_ROC_: 0.581, 95% CI: 0.533–0.630), or TG (AUC_ROC_: 0.545, 95% CI: 0.496–0.594) (Figure 4).

## Discussion

TGF-β1 is a multifunctional peptide that controls proliferation, differentiation, and other functions in many cell types, and it has dual regulation roles in immune response and cellular development. TGF-β1 acts synergistically with TGFα in inducing transformation. It also acts as a negative autocrine growth factor. Dysregulation of TGF-β1 activation and signaling may result in apoptosis. For example, TGF-β1 inhibits cancer cell growth at the early stage of tumor formation, and promotes the cancer development in the late phase [22].

Previous studies have shown plasma TGF-β1 levels to be reduced in patients with advanced atherosclerosis and angiographically proven CAD. The levels of TGF-β1 were thought to be inversely related to the development and severity of coronary disease [23-27]. By contrast, Border and Ruoslahti found that TGF-β1 could enhance atherogenesis by mediating excessive ECM deposition [28]. Another recent study revealed that a high plasma level of TGF-β had a significantly strong prognosis in terms of survival without cardiovascular events and survival without coronary interventions compared with the low TGF-β group (both p < 0.05), suggesting that plasma TGF-β may potentially have great prognostic value in patients with CAD [29]. Schaan et al maintained that serum TGF-β1 was not associated with CAD occurrence after clinical and laboratory evaluation of TGF-β1 in patients with CAD or DM [9]. However, they sampled fewer than 30 cases. We demonstrated significant positive correlation between serum TGF-β1/SMAD3 levels and CAD based on 547 subjects.

There has been no study stratifying patients according to CAD severity. It is probable that the relevance of TGF-β1 in CAD can only be detected in severe disease, or high concentrations of TGF-β1 from severe CAD may have a paradoxical effect [27,30,31].

In the correlation of TGF-β1 and SMAD3 levels with the risk factors of CAD, TGF-β1 is closely related to Lp(a) and uric acid, which both are considered to be markers of atherosclerosis, indicating that this cytokine may contribute to the establishment of CAD by regulating atherogenesis. SMAD3 levels correlated closely with blood glucose, implying that SMAD3 may influence CAD occurrence and the development of DM. Interestingly, it has been reported that risk factors including Lp(a), uric acid, BUN, triglycerides, and ApoA1 can be useful biomarkers for different clinical diagnoses [21,32], but we found that TGF-β1 and SMAD3 were more sensitive biomarkers for CAD than those factors mentioned above.

## Conclusion

Serum TGF-β1 and SMAD3 levels are closely associated with CAD. The underlying mechanism may be their regulatory effects on atherosclerosis and blood sugar.

A limitation of the present study is that the control population differs from the cases, in terms of age and cardiovascular risk factors. The mean age difference (~5 years) should be acceptable in an epidemiologic study of CAD according to a previous report [6,9]. CAD predominates in an older population.

## Competing interests

The authors had no conflicts of interest to declare in relation to this article.

## Authors’ contributions

WL and CC performed the study and wrote the manuscript. WC, JZ, and WL performed the study and/or contributed to data analysis and interpretation. XG, BL, and HW reviewed/approved the research protocol. CH takes full responsibility for the work as a whole, including the study design, access to data, and the decision to submit and publish the manuscript. All authors read and approved the final manuscript.

## Pre-publication history

The pre-publication history for this paper can be accessed here:

http://www.biomedcentral.com/1471-2261/14/18/prepub
